# Digesting the Role of JAK-STAT and Cytokine Signaling in Oral and Gastric Cancers

**DOI:** 10.3389/fimmu.2022.835997

**Published:** 2022-06-29

**Authors:** Yanhong Ni, Jun T. Low, John Silke, Lorraine A. O’Reilly

**Affiliations:** ^1^ Central Laboratory, Nanjing Stomatological Hospital, Medical School of Nanjing University, Nanjing, China; ^2^ Inflammation Division, The Walter and Eliza Hall Institute of Medical Research, Melbourne, VIC, Australia; ^3^ Department of Medical Biology, University of Melbourne, Parkville, VIC, Australia

**Keywords:** cytokines, JAK, STAT, oral, gastric, cancer, PROTAC (proteolysis-targeting chimeric molecule), TNF

## Abstract

When small proteins such as cytokines bind to their associated receptors on the plasma membrane, they can activate multiple internal signaling cascades allowing information from one cell to affect another. Frequently the signaling cascade leads to a change in gene expression that can affect cell functions such as proliferation, differentiation and homeostasis. The Janus kinase-signal transducer and activator of transcription (JAK-STAT) and the tumor necrosis factor receptor (TNFR) are the pivotal mechanisms employed for such communication. When deregulated, the JAK-STAT and the TNF receptor signaling pathways can induce chronic inflammatory phenotypes by promoting more cytokine production. Furthermore, these signaling pathways can promote replication, survival and metastasis of cancer cells. This review will summarize the essentials of the JAK/STAT and TNF signaling pathways and their regulation and the molecular mechanisms that lead to the dysregulation of the JAK-STAT pathway. The consequences of dysregulation, as ascertained from founding work in haematopoietic malignancies to more recent research in solid oral-gastrointestinal cancers, will also be discussed. Finally, this review will highlight the development and future of therapeutic applications which modulate the JAK-STAT or the TNF signaling pathways in cancers.

## 1 Introduction-JAK-STAT Signaling, Regulation and Associated Disease Susceptibility

Cells can communicate with each other and this is frequently accomplished by molecules, that are produced by one cell, then detected and interpreted by another. One class of these signaling molecules are secreted proteins, including cytokines, interferons and growth factors, otherwise known as ligands. These are detected by discrete receptor molecules which then initiate a cascade of signaling responses in the receptor cell that may result in, for example, small or large, acute or chronic (days to months) and reversible or irreversible changes (live or die) in the receptor cell. It is rare for particular receptor ligand combinations to work in isolation or to signal only one specific outcome, i.e. one signaling event may initiate multiple signaling branches that may in turn intersect, overlap or be affected by other signaling branches occurring concurrently. This makes analysis of signaling challenging but permits cells to respond with a degree of finesse to environmental cues.

The term cytokine encompasses interleukins, chemokines, interferons and tumour necrosis factors and all bind to corresponding receptors to activate cellular signaling pathways, including two related and evolutionary conserved signature signaling entities, the Janus Kinases (JAK: a family of tyrosine kinases) and the Signal Transducer and Activator of Transcription (STAT) protein family, which together constitute the JAK-STAT signaling pathway and has been studied in great detail (1–5). Another well studied pathway activated by cytokines is the NF-κB pathway, a family of inducible transcription factors, required in both innate and adaptive immunity to rapidly activate cellular responses (6, 7). Over 50 cytokines can signal through the JAK-STAT pathway, mediated by JAK-mediated phosphorylation, ultimately regulating a signaling cascade upstream of multiple cellular activities (8–13). The JAK-STAT pathway controls a multitude of biological processes; embryonic and immune-system development, stem cell continuity and innate and adaptive immune responses, inflammation and multiple aspects in the pathway to tumorigenesis (4, 13–15). JAK-STAT signaling therefore serves as a fundamental mechanism for how cells perceive and respond to environmental triggers and how they then further communicate their interpretation of these signals with other cells to control cellular fate outcomes. It is therefore not surprising that this pathway it is tightly regulated at multiple levels (5, 16), with aberrant activation due to dysregulated expression of cytokines or genetic mutations, gene amplifications, or gene polymorphisms resulting in chronic activation of this pathway. This is associated with a variety of human disease states, including; immunodeficiencies, interferonopathies, hematologic malignancies, inflammation, autoimmunity, the cytokine storm associated with COVID-19 mortality (17, 18), predisposition to bacterial and viral infections and, most relevant to this review, solid cancers (8, 19).

**Figure 1:**
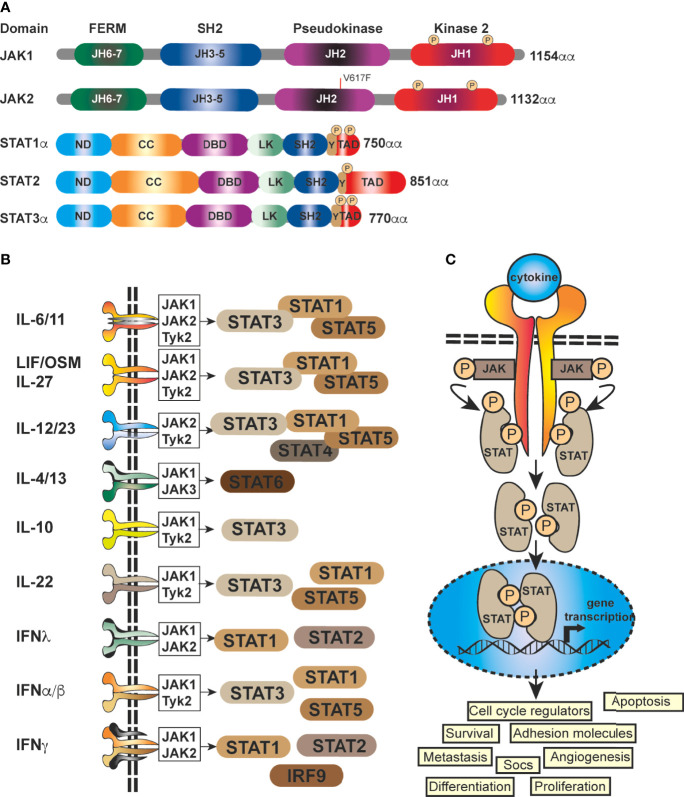
Schematic representation of the generic domain structure of JAKs and STATs. **(A)** Functional domains of the most important human JAKs and STATs regulating the progression of solid malignancies; ND, N-terminal; CC, coiled-coil; DBD, DNA-binding; LK, linker; SH2, Src-homology; TAD, transcriptional activation domain; JH1, kinase; JH2, pseudo-kinase domain with the ezrin, radixin, moesin (FERM) and SH2 domains forming a JAK receptor-binding module and the PK domain restrains Tyr kinase activity by binding the kinase domain. The common V617F mutation in JAK2 is shown (red line). **(B)** STATs are activated by a multitude of cytokines and IFNs. The most important ligands, receptors and pairing of JAKs regulating the progression of solid malignancies are shown. Shared receptor subunits are indicated by identical coloring. **(C)** Simplified schematic representation of the JAK-STAT signaling pathway. JAK activation occurs upon the binding of ligand and receptor multiprotein assembly, at which point two JAKs are brought into close proximity, permitting trans-phosphorylation. Once activated, the JAKs are capable of phosphorylating further targets, firstly the intracellular tails of the receptors on specific tyrosines, which then act as docking sites for their preferred substrates, the STAT proteins. Each STAT contains a conserved tyrosine residue near to the C- terminus transactivation domain (TAD), which is phosphorylated by the JAK proteins. This phospho-tyrosine promotes STAT protein dimerization *via* binding of an adjacent SRC homology 2, (SH2) domain and leads to exposure of a nuclear localisation signal (NLS), translocation into the nucleus, where they activate the transcription of genes involved in many cellular processes.

At it’s core, JAK-STAT signaling is quite simplistic, with a few elements; a cytokine, a receptor (cytokine receptor), a kinase (JAK kinase) and finally a transcription factor (STAT proteins) (5, 19), coupled with a regulator, the Suppressor Of Cytokine Signaling family of proteins (SOCS) (**Figure 1**). There are four mammalian members that belong to the JAK family; JAK1, JAK2, JAK3 and Tyk2, each of which selectively binds to different receptor chains, each comprising of seven JAK-homology (JH) domains (20) (**Figure 1A**). JAKs are capable of interacting with both types of cytokine receptors (I and II) (5), this includes receptors for interleukins, IFNs, and multiple others (21) **(** **Figure 1B**). As these receptors have no inherent enzymatic activity, preferential interaction with distinct JAK isoforms is required for their downstream signaling and STAT activation (5, 21, 22) (**Figure 1C**). The STAT family (STAT1–4, STAT5A, STAT5B, STAT6) is also structurally conserved (**Figure 1A**), but individual cytokine receptors preferentially activate distinct STATs (**Figure 1B**) through phosphorylation by the JAK proteins (3, 5, 23). This eventually leads *via* to exposure of a nuclear localisation signal (NLS) (**Figures 1A, B** **)** (1, 24). Within the nucleus, dimerized STATs can bind regulatory sequences such as GAS (Gamma interferon Activation Site) to control transcription of many target genes (8, 25, 26)] (**Figure 1C**) and integrate inputs from other signaling pathways, such as the NF-κB pathway.

**Figure 2:**
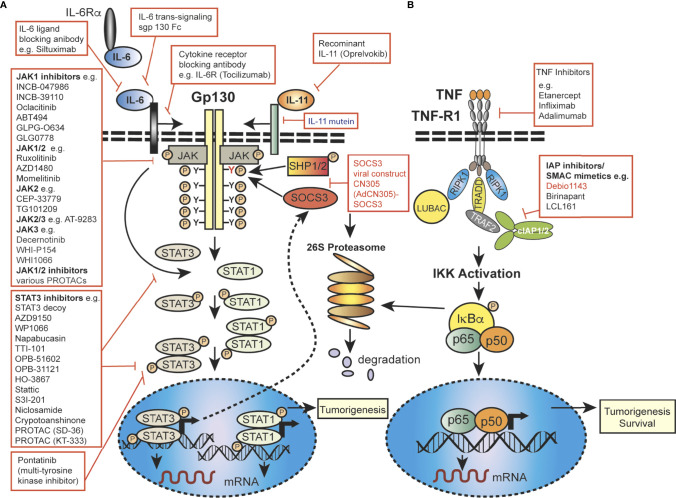
Mechanistic diagram of JAK-STAT and TNFR signalling and modes of inhibition. **(A)** The gp130 receptor complex and signaling pathways for IL-6 and IL-11 are shown as key activators of STAT transcription factors. The intracellular domain of the gp130 receptor contains a membrane-proximal tyrosine (Y; red), which provides a docking site for the suppressor for SOCSs proteins (e.g. SOCS3) and SHP2, while the membrane-distal tyrosine (Y) sites permits interactions with the SH2 domain of STAT1/3. The membrane proximal Y sites are phosphorylated by JAKs and upon phosphorylation of the distal Y site, STAT1 and STAT3 are recruited, homo-dimerise and are translocated into the nucleus, where they bind to specific target genes to regulate their expression. **(B)** Simplified schematic diagram of TNF/TNFR1 signaling pathway with downstream interacting proteins indicated. **(A, B)** Red arrows indicate intervention/inhibition points within each pathway and red boxes list examples of JAK-STAT or cytokine inhibitors/modifying drugs with their target protein.

Importantly, JAK-STAT signaling is controlled by the SOCS proteins, which function as part of a negative-feedback loop (19, 27, 28) and are rapidly upregulated upon activation of JAK-STAT signaling (29, 30). SOCS1 and SOCS3 are the most mechanistically and functionally defined members of this family and are potent inhibitors of the JAKs, using a range of mechanisms, such as phosphorylation inhibition, blocking STAT recruitment (30, 31) or binding to cytokine receptor complexes *via* (**Figure 2**) shutdown off JAK-STAT signaling (21, 27). JAK activity is not only regulated by SOCSs proteins but also by protein tyrosine phosphatases (PTPs) (5, 19). STAT proteins are also controlled at multiple levels, and in addition to ubiquitin-mediated degradation, they can be inhibited by, for example, PIAS (Protein Inhibitors of Activated STAT) in the nucleus (32). In addition to the endogenous control of the JAK-STAT pathway, this signalling cascade can be modulated by various mechanisms, including autocrine/paracrine cytokine production, JAK protein mutation, upstream cancer-causing genes activating STATs, or more rarely STAT mutations themselves, possibly resulting in continuous pathway activation. Mutations and polymorphisms which dysregulate the JAK-STAT pathway can result in a variety of human conditions, such as inflammatory related diseases, an array of leukemias and even solid cancers (8, 15, 33–36).

JAKs are vital components of the JAK-STAT transduction pathway and their influential role in governing cellular survival, proliferation, differentiation and apoptosis is well studied (19, 37). Extensive clinical and genetic studies in human and in murine models have established a role for constitutively activated JAKs in human inflammatory/autoimmune diseases, myeloproliferative diseases and select cancers (8, 12, 19, 34, 38–43). For example, the common V617F mutation (**Figure 1A**), which results in constitutively activated JAK2, is associated with multiple disease syndromes. including elevated erythrocytes and megakaryocytes. This underlines the importance of JAK signaling in immune cell activities and haematological malignancies and provides the starting point for investigations into solid tumours. The generation of knockout or conditional knockout mice for each JAK family member has also provided an understanding of the interconnectedness between JAKs and their cognate cytokine receptors and the existence of restricted preferential relationships between cytokine receptors and their downstream JAK effectors, paralleling the phenotype of inactivating JAK mutations in humans (reviewed (12, 19, 44–47). However, functional redundancy exists, whereby in the absence of a single JAK, another JAK family member can fulfil the same signaling function (4, 19) (**Figure 1B** **).** In addition, the idea of hierarchical role for JAKs, with one JAK family member functioning as the upstream activator of a neighbouring heterotypic JAK family member within a cytokine receptor complex is another factor contributing to their functional complexity [reviewed (19)]. JAK1 and JAK3 GOF mutations occur in human leukemias and multiple myelomas (34, 48) but also in solid cancers, including breast, gastric, colon, lung and hepatitis B associated hepatocellular carcinomas, particularly in the pseudokinase domain and adjoining linker region (**Figure 1A**) (8, 16, 19, 34, 35). These clinical and genetic studies in human and in murine models have established a role for constitutively activated JAKs in human inflammatory/autoimmune diseases, myeloproliferative diseases and select cancers. Unsurprisingly, this role for activated JAKs in human disease has instigated immense interest by pharmaceutical companies to develop JAK inhibitors (15).

In recent years it has become apparent that the STAT proteins are linked to a variety of normal biological functions and these include cell differentiation, cell cycle control and proliferation, cell death and various aspects of the immune response. Mutations in STAT1, -2, -3, -5B, -6, while rare are associated with multiple inherited conditions, particularly primary immunodeficiencies, autoimmune diseases and even cancers (4, 8). Ultimately, the type of disorder developed is dependent on how STAT signaling is affected and whether the mutation is a GOF or LOF STAT gene defect. Seven inherited disorders are known to be caused by mutations in the STAT family of genes (49), with heterozygous STAT1 GOF mutations linked to increased susceptibility to certain infections, autoimmunity and risk for tumor development, while STAT2 insufficiency is characterized by susceptibility to multiple types of viral infections. Signaling through STAT3 is mediated through multiple receptors (**Figure 1B**), therefore a range of immunological and phenotypic manifestations is to be anticipated (8, 49). The contribution of specific STAT family members to control of normal cellular processes has also been evaluated by homozygous deletion or conditional knockouts. Probably the most important take home message is the establishment of a non-redundant role for STAT1 protein in IFN signaling (50), while STAT2 and STAT3, homozygous deletion resulted in embryonic lethality (51, 52). Conditional deletion in mice (53) subsequently established that STAT3 plays a crucial role in the signal transduction of various cytokines, including IL-6 (53). Thus, murine STAT proteins, like their human counterparts have diverse effects in response to extracellular signaling proteins, achieved by altering gene transcription in the effector cells.

In terms of human disease, the extended STAT family are also implicated in many aspects of tumorigenesis in humans as well as resistance to chemotherapy treatments. The most habitually mutated gene from this family in haematopoietic cancers is STAT3 [reviewed (4, 54, 55)] and to a lesser extent STAT5B (4, 22, 54). Somatic STAT3 mutation also plays a crucial role in select solid cancers, including skin cancers, GI and neural tumors, while STAT1, -2, -4 and -6 appear to have more limited roles in tumorigenesis (22). The majority of these diseases result from the consequential dysregulation of this signaling pathway, particularly related to JAK family member mutations, but mutation of STATs is not without clinical consequence (56). While mutations in members of the JAK-STAT pathway play a role in the development of a variety of diseases, particularly immunodeficiency syndromes and predisposition to infections, inflammatory processes themselves are one of the major drivers of tumor initiation, progression and metastasis (57, 58). This is in part driven by pro-inflammatory cytokines binding to their cognate receptors resulting in aberrant and often increased activation of JAK-STAT or TNF signaling (**Figure 1C**), although the exact mechanisms driving the penultimate stages of oncogenic invasion and metastasis are unknown (8). These tightly regulated STAT signaling control mechanisms can be disrupted in cancer cells, altering an otherwise finely-tuned homeostatic balance, which also occurs in GI cancers including Oral Squamous Cell Carcinoma (OSCC) (59), which comes under umbrella term of Head and Neck Squamous Cell Carcinomas (HNSCC) and Gastric Cancer (GC) (37, 60). Dysregulated cytokines levels are a hallmark of many gastrointestinal cancers and this review will focus on the role of JAK-STAT and introduce the TNF signaling pathways. Finally, we will discuss how current therapies and those in development, (eg. cytokine immunotherapy, Smac Mimetics and PROTACs) may be used modulate these signature pathways with the aim to increase curative rates for these low survival cancers.

## 2 Cytokines, JAK-STAT Activation and Inflammation

As outlined above, constitutive triggering of the JAKs and STATs through elevated cytokine levels is linked to many chronic inflammatory diseases (61, reviewed; 57, 62). Such chronic inflammation may assist in promoting the cellular proliferation of nascent tumor cells, transformation and metastasis or conversely constraining the anti-tumor response. Cytokines also act on epithelial cells lining the gastrointestinal tract and other cell types to regulate secretion, proliferation, and differentiation (63). Inappropriate cellular epithelial activation may be one such consequence but may be quite diverse in different solid cancers (15, 16, recently reviewed 57, 64). Cytokines and their receptors are also polymorphic, and subtle genetic variations or single nucleotide polymorphisms (SNPs) predominantly found in the promotor region of genes such as IL-1β, 1-Rα, -2, -6, -10, -12, -13, -16, -18, TNF, IFN-γ, TGF-β are linked to functional changes and correlate with increased susceptibility to infections, autoimmunity, certain cancers and their differing treatment outcomes (65–67). Cytokine production by tumour infiltrating cells (TILs) or stromal cells such as endothelial cells or fibroblasts within solid tumor microenvironments (TME) mediate communication between tumor and TILs (57, 62, 68, 69). For example, chronic STAT3 signaling by cytokines such as IL-6 in transformed cancer cells can induce cell proliferation and activation of MMP’s (matrix metalloproteinases), thereby promoting tumor invasiveness and EMT (epithelia-to-mesenchymal transition) and expression of “master” EMT transcription factors including Twist and Snail (70). In addition, STAT3 becomes phosphorylated (hyperactivated) not only in the tumour cells but also in immune cells and CAFs within the TME (71), which could impact anti-tumor immunity. Chronic STAT3 activation is also associated with the elevated expression of factors promoting cell cycle and cell survival (cyclin D1, survivin and Bcl-xL) (72). Dysregulated cytokine expression levels and JAK-STAT signaling are also hallmarks of oral and gastric cancers (73). Activation of STAT3, STAT5B or JAK2 due to mutational changes are associated with certain heamatopoietic cancers and solid cancers (gastric cancer, breast cancer, lung cancer and oral cancer), while *STAT3* and *STAT5B* are also considered to be bona fide oncogenes, since constitutively-activated forms induce cell transformation and invasion of cancer cells in mice (74–79). However, JAK-STAT signaling is complicated and often has context dependent effects. In certain situations and in certain cell types, it may instead have a tumor suppressive role, as is the case for STAT1 (80) and for STAT5, which may inhibit tumor progression in the liver but also acts as a tumor suppressor in fibroblasts (reviewed 76).

### 2.1 The Role of Cytokines and JAK-STAT Signaling in Oral Cancer and Gastric Cancer

Several cytokines are particularly relevant to oral cancer and GC, such as those having proinflammatory functions; IFNγ, IL-6, IL-11 and other cytokines which are predominantly anti-inflammatory, such as transforming growth factor beta-1 (TGFβ), IL-4, IL-10 and IL-13 which all signal through the JAK-STAT pathway, except TGFβ, which signals through a receptor tyrosine kinase (57, 62). IL-6 mediated JAK-STAT signaling is required for normal homeostatic processes and is kept in check by the restricted pattern of expression of IL-6Rα to a subset of leukocytes and hepatocytes (81). However, trans-signalling through a soluble form of the IL-6Rα released from cells by proteolytic cleavage permits the formation of a IL-6/sIL-6Rα complex, which binds in *trans* to activate membrane-anchored gp130 (**Figure 2A**) (81). It is this latter signaling that appears to be involved in the inflammatory response and relevant to this review is involved in inflammation driven tumor response and T cell proliferation, leukocyte recruitment and activation of stromal cells (57, 82). Within the tumor microenvironment IL-6 is produced by a multitude of cell types including tumor cells, immune cells, and stromal cells. Elevated triggering of STAT3 by dysregulated IL-6 combined with additional oncogenic driver mutations, such as in *KRAS* or *TP53*, can drive tumor development in the oral cavity (83) and stomach (84).

### 2.2 TNF Signaling; A Common Activation Mechanisms for Cancers

In addition to cytokines which signal through the JAK-STAT pathway, initiation and development of inflammation driven cancers can also be driven by the master proinflammatory cytokine Tumor Necrosis Factor (TNF), an important regulator of the immune response in both the steady state and in disease processes and a critical mediator of carcinogenesis, as regulator of cellular proliferation, invasiveness and metastasis of a multitude of cancers (85, 86). Macrophages and T-cells are major sources of TNF, but other cells such as B-cells, endothelial cells and neutrophils also produce it (86, 87). TNF binding to its receptors TNFR1 and TNFR2, can cause a dizzying range of effects facilitating TIL invasion of tumors and promoting angiogenesis and tumour cell migration and invasion, but also promoting cell survival or cell death (88, 89). TNF is expressed as a type II transmembrane protein but is also liberated into a soluble form by cleavage by a membrane associated metalloproteinase, ADAM17 also known as TNF Converting enzyme (TACE) which also, incidentally, liberates IL6R for trans-signalling (90). Both forms of TNF interact with TNFR1 and TNFR2 but while membrane TNF stimulates both TNF receptors, soluble TNF largely fails to stimulate TNFR2 despite high-affinity binding (91). TNFR1 is widely expressed on most cell types and stimulates transcription of a host of inflammatory mediators including other cytokines by activating transcription and stabilising mRNA in a Nuclear factor of kappa B (NF-κB) (**Figure 2B**) and MAP kinase dependent manner (91, 92). However, in certain circumstances, particularly where transcription or signalling is interfered with, TNF can also induce cell death. In general, TNFR2 has a more restricted expression pattern and is predominantly expressed in myeloid cells and regulatory T-cells. TNFR2 has no intrinsic cell death inducing mechanism but stimulates NF-κB signaling and activation of various kinases and, while it cannot directly induce cell death it can switch TNFR1 signalling to cell death (91, 93). Activation of the TNF/TNFR2 pathway has been established as a critical bio-marker of several cancers including oral (94) and gastric cancers (63, 95).

## 3 Oral Squamous Cell Carcinoma

**Figure 3:**
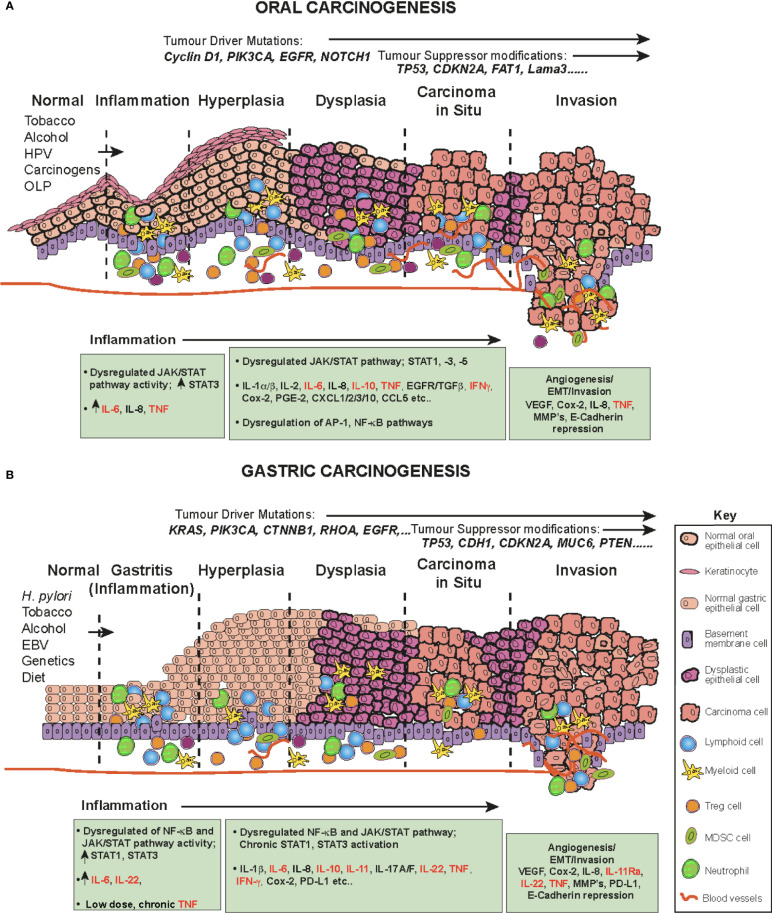
The role of cytokines and JAK/STAT in OSCC and GC in a step-wise model. The normal epithelium (left) progresses to **(A)** OSCC or **(B)** or GC, through a series of histopathological precursors. During early disease stage the immune system is activated by inflammatory stimuli, resulting in both pro-tumor and anti-tumor effects mediated by inflammatory cells. The cytokine and JAK-STAT pathway are dysregulated within the cells of the inflammatory microenvironment and target genes serve as fuel to control both apoptosis and inflammation. Pro-inflammatory cytokine mediators promote neoplastic growth and development, proliferation, tissue remodeling (EMT), angiogenesis and metastases. During this progression, several alterations in key genes also accumulate (e.g. *KRAS*, *TP53*) to progress tumorigenesis.

Oral squamous cell carcinoma (OSCC) is one of the most common human malignancies and a leading cause of morbidity and mortality world-wide, constituting 4% of all systemic malignant tumors (96–98) OSCC also constitutes a major subcategory of HNSCC. These cancers originate in the oral and oropharyngeal subsites (tongue, lips, gingiva and retromolar trigone) and account for approximately 90% of all oral cancers (96). The oral cavity is the first station of digestive tract to be exposed to a multitude of environmental stimuli, including viral and bacterial infections and continuous chemical irritation. The main risk factors for OSCC are entirely predictable and include; tobacco smoking, alcohol consumption, continuous mastication of Areca-nut/betel-leaf/tobacco-quid (particularly in Southeast Asia), infection with human papillomavirus (HPV) and the inflammatory autoimmune condition Oral Lichen Planus (OLP, especially the erosive form), a possible precursor lesion of OSCC (96, 99, 100). These carcinogens and inflammatory agents and conditions contribute to a progressive influx of inflammatory cells (CD4 and CD8 T cells, B cells, NK cells, neutrophils, eosinophils, macrophages and plasma cells (101)), resulting in the accumulation of genetic and epigenetic lesions throughout the oral mucosa, affecting cell cycle, DNA repair mechanisms, cell differentiation and apoptosis and resulting in the transformation of normal keratinocytes to hyperkeratosis, oral dysplasia, the development of carcinoma *in situ*, then invasive cancer (102) (**Figure 3A**). Indeed, the oral cavity may be carpeted with pre-cancerous lesions with a high risk for malignant transformation (96). During the preceding period, chronic inflammation, driven in part by dysregulated salivary cytokines, a hallmark of OSCC, drives and maintains neoplastic transformation; a highly complex multifactorial process occurring in epithelial cells through the premalignant lesions, leukoplakia and erythroplakia to epithelial-to-mesenchymal transition EMT and eventually invasive cancer (**Figure 3A**) (94, 96, 100, 103). A direct link between inflammation in the oral cavity and promotion of tumor invasion has been established (1, 94, 104), in which elevated cytokines (94) and dysregulation of the JAK-STAT signaling pathway have been implicated (105, 106).

Certain syndromes such as Fanconi anemia or Dyskeratosis congenita can predispose to cancer of the oral cavity, however, these conditions are rare and usually OSCC occurs spontaneously in aged individuals (107). The associated accumulation of genetic and epigenetic alterations due to chronic inflammation results in oncogenic activation and inactivation or loss of tumor suppressor genes. These include frequent DNA copy number gains at chromosomes 3q, 5p and 8q and copy number losses on 3p and 8p (108). In addition, several driver/tumor suppressor modifications have been identified in OSCC, including; *TP53*, *NOTCH1*, *EGFR* (epidermal growth factor receptor), *CDKN2A* (cyclin-dependent kinase inhibitor 2a), Cyclin D1, Rb (retinoblastoma), *FAT1* and *Lama3* (105, 108–113) (**Figure 3A**).

Despite recent advances in clinical diagnosis and improvements in systemic therapeutics (cisplatin, 5FU (5-fluorouracil), docetaxel) and radiotherapy, the 5-year survival for HNSCC has remained at ~50% for the last 30 years for regional and more disseminated disease (99, 104). In addition, both the cancer and its treatment cause considerable morbidity with acute and long-term toxicity impacting speech, swallowing, nutrition and appearance (114, 115). The difficulty with early diagnosis and the high potential for neck lymph node metastasis with invasive properties continues to account for the high mortality (103, 116).

### 3.1 Dysregulation of Cytokines as Promotors and Mediators of OSCC

Potential novel biomarkers to identify high risk disease individuals, such as activating JAK mutations have been detected in some solid tumors, such as gastric (77) and for both JAKs and STATs in haematological malignancies (4, 8). However, these haven’t been reported to be associated with OSCC (117). Tests of predicted functional polymorphisms in HNSCC for *SOCS3*, have been conflicting; rs2280148 located at the 3′-untranslated region, indicated a predicted increase in the risk for this cancer, while rs8064821 located in the promoter region was associated with an decreased risk (118). Functional polymorphisms affecting gene expression of IL-4,-6,-8,-10 and TNF have been shown to strongly associate with increased risk for oral cancer (119). Single nucleotide polymorphisms (SNPs) in the promoter region of the TNF gene are linked to HNSCC susceptibility. For example, the TNF-308 position (AA/GA haplotype) SNP was shown to increase TNF expression and contribute to greater malignant clinical aggressiveness and reduced overall survival (120). More recently, patients with the CC genotype of TNF–1211, were shown to have higher plasma TNF associated with more severe oral mucositis complications and reduced survival when administered with chemoradiotherapy (121). On the other hand, patients with the TNF−238 A/G SNP homozygous recessive allelic variant had a reduced risk of oral precancer (122). Overall, these genetic association studies addressing the predisposition to oral cancer, highlight susceptibility differences at the population level, possible predictable treatment outcomes of these cancers and novel target avenues, which may be tested in animal models and are discussed later in this review.

Cells of the upper aerodigestive tract epithelium, are regulated, in part, by multiple cellular growth factors and OSCC demonstrates inappropriate activation of JAK-STAT signaling, particularly STAT3 but also STAT-1A, 5A and -5B (102, 123–125) as discussed in a number of recent comprehensive reviews (72, 117). However, the mechanistic links of the JAK-STAT pathway in OSCC are still being investigated. Inflammatory cytokines and chemokines, which activate the JAK-STAT pathway have been identified as early bio-markers and mediators of OSCC [recently reviewed (94)]. Gene expression microarray analysis of OSCC cell lines with high NF-κB activity and OSCC patient samples identified dysregulation of genes involved in inflammation, wound healing, angiogenesis and growth regulation and upregulated levels of IL-8, CCL5, STAT1, and VEGF in OSCC (102). In this review we primarily summarize the role of the cytokine IL-6, which signals through JAK1/JAK2 to activate STAT-1,-3,-5 (**Figures 1B**, **2A**) and TNF (**Figure 2B**), since these cytokines play major roles in OSCC initiation and development of OSCC (94) (**Figure 3A**).

In general, the early phase of OSCC is asymptomatic, accounting for both generalized late detection and diagnosis (97). The identification of early OSCC biomarkers is of great importance to improving survival rates (126), because early detection dramatically improves survival but unfortunately it is exceptionally difficult and lesions are often overlooked (127). To address this problem, non-invasive salivary secretion diagnostics, termed a “liquid biopsies”, are being explored. TNF has been shown to have role in the progression of OLP and meta-analysis of multiple studies has shown higher levels of TNF in salivary secretions (128). Initial studies indicated that it was possible to distinguish cancer from non-malignant lesions. IFN, IL-1β, IL-2, IL-6, TNF and IL-8 were amongst the most commonly studied saliva cytokines, with elevated levels possible predictors of early malignancy [reviewed (129–131)]. However, differentiating between inflammatory conditions, such as periodontitis and OSCC is difficult (130). More recently several systemic and qualitative reviews from meta-analyses of OSCC case-controlled studies identified IL-2, IL-6, IL-8, TNF, IL1β and IL-10 as being significantly upregulated in OSCC patient saliva by ELISA (129, 132, 133). Except for IL-10, these cytokines were also higher in OSCC patients and oral, potentially malignant, disorders such as leukoplakia when this latter group were compared to healthy controls (132). Overall, the most promising and reliable diagnostic cytokines for OSCC, significantly elevated from other oral potentially malignant disorders from these studies are IL-6, IL-8 and TNF (101, 132, 133) (**Figure 3A**). While this early screening approach is promising, there are still many issues to be tackled before it can be adopted clinically for early diagnosis screening of high-risk populations.

Saliva cytokine levels of IL-1β, IL-2, IL-6 and TNF are not only associated with oral inflammation but also with the severity of oral mucosal damage in cancer patients. For example, a literature analysis by Uz et al. suggested that overexpression and elevated serum and/or saliva IL-6 concentrations in patients with HNSCC are related to poor survival and increased tumorigenicity (134). TNF also plays additional roles in promoting tumorigenesis and is overexpressed in human oral cancer tissues (135). It is also associated with pain, albeit in murine studies (136). However, HNSCC are a group of cancers associated with a high prevalence of pain (137) and pre-treatment for pain may serve as a prognostic factor for patient survival (138). *In vitro* studies also suggest that TNF enhances the invasion and metastasis ability of OSCC cell lines *via* the NF-κB signaling pathway (139). In addition, while it is believed that HPV infection alone may be insufficient for the oncogenic transformation of normal epithelial cells recent studies suggest that chronic exposure of TNF to HPV-infected oral keratinocyte cell lines increases cancer stem cell-like populations and stemness (140). This would suggest that HPV and TNF may act in concert to promote oral malignant conversion and that mechanisms to inhibit TNF may be of benefit for OSCC.

### 3.2 Dysregulation of the JAK-STAT Pathway in OSCC

The 2015 TCGA study of head and neck cancers (constituting 62% oral cancers) identified amplifications in several oncogenes including the JAK-STAT linked receptor tyrosine kinases, *EGFR* and *IGF1R* (105). Nuclear and cytoplasmic interactions between EGFR and STAT3, increase the expression of several EMT promotors (iNOS, cyclin D1, c-fos) through direct binding of the EGFR/STAT3 complex to their promoters [reviewed (141)] resulting in consistent JAK-STAT triggering in HNSCC (4) (**Figure 3A**). IGF1R is also an upstream signal for JAK-STAT activation, particularly STAT3 (141).

Both HPV^+^ and HPV^-^ HNSCCs demonstrate aberrant regulation of JAK-STAT signaling, with upregulation of STAT3 and its many gene targets contributing to malignancy and therapy resistance *via* cell cycle, cell growth and inhibition of cell death mechanisms (72, 73, 117, 142) (**Figure 3A**). Inhibition of aberrant STAT3 activity has been shown to impede OSCC growth both *in vitro* and *in vivo*, (72), and, coupled with the association of STAT3 hyperactivation with poor prognosis, resistance to standard therapies, and immune escape, this makes it a potential therapeutic target for OSCC (72, 102). IL-6 is one of the key upstream cytokines implicated in poor clinical outcomes in OSCC patients (143). STAT3 activity is controlled in part by the activity of the prototypical pro-inflammatory IL-6/gp130 signaling cascade (144) (**Figure 2A**). This STAT3 activation can in turn lead to elevated IL-6 expression creating a positive feedback loop that fuels the creation of an inflammatory and a pro-tumorigenic milieu (144). Multiple STAT3-controlled signal transduction pathways are associated with OSCC/HNSCC. In addition growth factors including EGF, TGF-α and platelet-derived growth factor can activate members of the STAT family, including STAT1 and STAT3 (145). Overexpression of EGFR and its ligand TGF have been detected in tumors and cell lines established from OSCC patients and EGFR protein is required to sustain OSCC cells *in vitro* (146). Stimulation of TGF/EGFR activates both STAT1 and STAT3, however, this TGF-α/EGFR mediated autocrine growth of transformed OSCC epithelial cells appears to be reliant on activation of STAT3 but not STAT1 (123).

In solid tumors, STAT1 is generally considered as a tumor suppressor but there is growing evidence that STAT1 also has a pro-tumorigenic function, perhaps in a cell-type specific context (80). The role of STAT1 in OSCC is controversial (117). Previous studies have shown that in chemotherapy treated OSCC patients, increased levels of p-STAT1 had a positive prognostic association, with a predictive increase in overall survival (147, 148). However, another more recent study showed the opposite correlation, with higher intra-tumoral p-STAT1 associated with worse survival (149). Despite these contradictory studies, microarray analysis of OSCC transcriptomes recently suggested that STAT1 is an important contributor to OSCC development, and that STAT1 might therefore serve as a potential diagnostic biomarker (150). One issue is that the role of STAT1 in OSCC may not actually correlate with expression levels within the tumor itself, but rather with the immune cells constituting the inflammatory tumor milieu. For example, in pre-malignant OSCC, CD163^+^ TAMs (tumor associated macrophages) are the main cells that express STAT1 and p-STAT1 (151). Relevant to both OSCC and EBV^+^-gastric cancer, JAK2/STAT1 signaling has been shown to mediate respectively EGFR- or IRF-1, IFNγ-induced upregulation of the programmed cell death-ligand 1 (PD-L1), an inhibitor of T-cell-mediated tumor cytotoxicity (152, 153) as discussed later in this review.

STAT3 transforms human epithelial cells and has been defined as an OSCC oncogene and identified as a negative prognostic factor in human OSCC (154). A constitutively activated JAK-STAT pathway, particularly STAT3 is an early event in OSCC (124) and shown to be mediated by the autocrine/paracrine stimulation of the IL-6/gp130 cytokine/receptor (155). In addition to being a core early event in OSCC, abnormal STAT3 activation represents a potential risk factor for poor prognosis in early-stage patients (106, 124) and in later disease stages correlates with poor tumor differentiation, lymph node metastasis and reduced survival (16, 106). Expression of a dominant-negative mutant STAT3 in HNSCC/OSCC cell lines has been shown to prevent proliferation, trigger apoptosis, and inhibit the downstream pathways associated with STAT activation (124). Thus, STAT3 activation is a fundamental underlying factor in a multitude of malignant behaviours in OSCC, contributing to cell proliferation, differentiation and apoptosis resulting in neovascularization, establishment of a pro-inflammatory state. Hyperactivation of STAT3 is also implicated in both treatment resistance and immune escape within the oral cavity (72, 123, 124, 156). In addition, LOF protein tyrosine phosphatase receptor type T (PTPRT) by somatic mutation or promoter hypermethylation (31-60% of HNSCC), increases STAT3 activation and sensitivity to STAT3 inhibition (72, 157). Tumor suppressors that dephosphorylate STAT3, may also lead to prolonged phosphorylation and activation of STAT3 (72, 105, 157). Dysregulation of STAT3 and enhanced expression of the active phospho-form not only within the tumor cells themselves but also within the TILs, including fibroblasts constituting the tumor microenvironment can support solid tumor growth (158). Therefore, STAT3 activation most likely engages the communication between these types in the OSCC microenvironment promoting tumor progression. In addition to serving as an oncogene in OSCC, STAT3 is a resistance mechanism for standard chemotherapeutics and radiation, the current treatment modalities for this cancer (99).

Cancer-associated fibroblasts (CAFs), are major cellular components of the OSCC stroma and communicate with tumor cells to stimulate cancer cell growth, survival, and invasion and associated with poorer survival outcomes. Epiregulin (EREG) a member of the Epithelial Growth Factor (EGF) family promotes tumor development, migration and invasion (159). We recently showed that EREG is upregulated in CAFs from OSCC patients, with elevated expression correlating with the tumor severity and predicted shorter overall survival (160). Mechanistically, EREG appeared to be essential for normal fibroblast to CAF transformation and essential for the induction of tumor cell EMT in a JAK2-STAT3- and IL-6-dependent manner (160). This recent finding further underlines the importance of the JAK-STAT pathway in the progression of OSCC by activating genes important in the initiation of tumor metastasis.

Compared to STAT3, STAT5 activation plays a relatively minor role in OSCC/HNSSC but STAT5 can contribute to the development of tumorigenesis and increased expression of STAT5 proteins and phospho-STAT5 has been demonstrated in these tumors (161). SOCS2 protein is significantly downregulated in OSCC patients, and its levels are inversely correlated with miR-424-5p expression, through a newly described IL-8/miR-424-5p/STAT5 pathway in OSCC (162). This pathway involves the pro-inflammatory cytokine IL-8 activating STAT5, which then induces SOCS2 (a STAT5 inhibitor) and miR-424-5p. MiR-424-5p expression however suppressed SOCS2 activity and led to constitutive STAT5 expression. Such elevated STAT5 expression correlated with increased tumor cell migration and invasion through elevated matrix metalloproteinase activity in OSCC cancer cells. Copy number variations were increased in OSCC when compared with normal or pre-malignant oral lichen planus samples. In 7/15 samples there was an increase in chromosome 9 sequences in a region which encompass *JAK2*, as well as 38 other genes (163).

### 3.3 Lessons From Animal Models of OSCC

Clinical and descriptive studies of biopsy samples and analysis of cell lines from patient OSCC, are informative for later disease stages but rarely provide insight into disease development. Animal models of chemical induced oral carcinogenesis and transgenic animals are useful in this regard and also to assess therapeutic approaches and the impact of the immune system (103). The most frequently used chemicals are DMBA (9,10-dimethyl-1,2-benzanthracene) and 4NQO (4-nitroquinoline-1 oxide). DMBA is highly irritating and produces an inflammatory response and necrosis. 4NQO, acts similarly but is more efficacious in inducing tumorigenesis and has been shown to mimic the process of oral carcinogenesis in humans (113, 124, 164, 165). These murine cancers, share pathologic and biochemical features with tobacco-related human OSCC, including epidermal growth factor receptor (EGFR) overexpression downregulation of p16 (164), elevated STAT1 (166), STAT3 (164, 165), overexpression of SOCS1, -3 (167), elevated pro-inflammatory cytokines (IL-1β, IL-6, TNF) and MMPs (136, 168). Furthermore, as we have shown, loss of NF-κB signalling accelerates tumorigenesis (169). Regulatory T cell (Tregs) enrichment and function also appears to be modulated by STAT3 in response to radiation therapy in a murine oral orthotopic model with DMBA induced HNSCC/OSCC (170). Since radioresistance is a major issue with human HNSCC, this suggests that STAT3 inhibition may benefit patients receiving this type of therapy.

Moreover, the mutational spectrum of human OSCC was shown recently to be replicated in the 4NQO mouse model, including mutations in *Tp53*, *PIK3ca*, *Notch1*, *Fat1* and *Lama3* with gene ontogeny analysis identifying cytokine signalling as a major biological process linking the human and murine disease (113). In addition, a cross-species genomic comparison of DMBA carcinogen-induced murine and human OSCCs with indolent or metastatic growth using next gen sequencing also revealed conservation of the human driver pathway mutations in mouse OSCC; *Tp53*, *Mapks*, *Pi3k*, *Notch*, *Fat1–4* and the *Jak*-*Stat* pathway (171). There is an opportunity to delve more deeply into the roles of these factors in initiation and development of oral cancer because not many studies have used genetically modified mouse models deficient in cytokines or elements of the JAK-STAT pathway to explore their roles in these chemical induced OSCC models (169).

## 4 The role of Cytokines and JAK-STAT Signaling in Gastric Cancer

### 4.1 Gastric Cancer

The stomach is the next station in the alimentary tract down from the oral cavity and aids in the adsorption of nutrients by secreting hydrochloric acid and enzymes. As with the oral epithelium, the gastric epithelium is exposed to multiple exogenous stimuli, which can result in chronic inflammation. In most human GCs, this inflammation is initiated by infectious agents, such as *Helicobacter pylori (H. pylori)* or Epstein-Barr virus (EBV) infection or prolonged exposure to gastric irritants, such as a diet high in salt and nitrated foods (172) (**Figure 3B**). These agents promote chronic inflammatory gastritis, subsequently leading to pre-cancerous alterations in the stomach epithelial lining, which combined with aging may have an additive role in the promotion of GC (63, 172–174) (**Figure 3B**). Gastric cancer (GC) is the 5^th^ most common human cancer that imposes the 4^th^ highest cause of cancer mortality world-wide (98). This is in part due to the frequently asymptomatic nature of this disease which often results in a late-stage diagnosis with locally advanced or metastatic disease and limited curative opportunities (172). More than 90% of gastric cancers (GCs) are adenocarcinomas, which originate from epithelial cells in the chronically inflamed gastric mucosa. A pathology based, classification of GC identified diffuse, intestinal or mixed sub-types (175), which have been subsequently divided into four molecular sub-types; genomically stable (GS), microsatellite instability (MSI), EBV^+^, and a Chromosomal instable type (CIN) (77). A small proportion of chronic gastritis patients develop gastric cancer, suggesting additional risk for genetic and environmental factors in GC development (172). DNA polymorphisms that increase GC risk have mapped to genes encoding cytokines (63). Many cytokines secreted by immune cells and epithelial cells during chronic gastritis have been linked to poor patient outcomes (176).

GC usually occurs spontaneously with only about 8-10% of cases due to inherited mutations, such as in E-cadherin (*CDH1*) resulting in an autosomal dominant GC predisposition (177). Gastrointestinal polyposis syndromes can also predispose to GC (178). Multiple studies including the Cancer Genome Atlas Research Network (77), in addition to the risk factors described above, have revealed certain genetic alterations and/or mutations can contribute to GC pathogenesis including; *PIK3CA*, *TP53*, *KRAS*, *APC*, *STK11*, *CTNNB1*, *CDKN2A*, *ARID1A*, *ERBB2, FGFR1, FGFR2, EGFR* and *MET* and (**Figure 3B**) (77). A novel recurrent amplification at 9p24.1, the locus containing *JAK2*, *CD274* (PD-L1) and *PDCD1LG2* (PD-L2) were also described in the EBV^+^ GC subtype (77, 179, 180). *CD274* (PD-L1) and *PDCD1LG2* (PD-L2) are involved in immunosuppression by tumor cells to evade cytotoxic T cell mediated killing and are associated predominantly with the EBV^+^ sub-type (77) in a STAT1 driven manner as discussed later in this review (153).

Hopes that anti-inflammatory drugs and *H. pylori* eradication during early disease stages may prevent disease progression, have not been entirely realised (181) but may reduce risk (182). However, current treatments (chemotherapy, radiation, surgical resection) are often given with palliative intent. High rates of relapse for GC are indicative of the failure to address the chronic inflammation that is believed to one of the critical disease drivers. Both the NF-κB and JAK-STAT pathways are known to promote inflammation-associated tumorigenesis within the GI tract (7, 60) and deregulation of these pro-inflammatory signaling pathways, results in gastric intestinal dysplasia and eventually, over decades results in invasive adenocarcinoma (173). Significant improvements in overall survival rates of patients, which currently stand at 30% 5 years post diagnosis have not, to date, been achieved (98).

### 4.2 Inflammatory Cytokines Dysregulates the JAK-STAT Pathway in Gastric Cancer

*H. pylori* is the leading risk factor for GC, infecting the stomach lumen. In most cases the immune response is unable to clear the infection and the inflammation becomes chronic, creating molecular and cellular changes favouring the transition to tumorigenesis (174). However, this response is variable and is dependent upon both host genetics and *H. pylori* strain (183). Activation of inflammatory genes and elevated pro-inflammatory cytokines including IL-1β, IL-1Rα and the neutrophil attracting cytokine IL-8, IL-10, -11, -17A, -17F, -22 and TNF (reviewed (63, 84, 184) increase the risk for atrophic gastritis and GC (174) (**Figure 3B**). Many of these cytokines activate the JAK-STAT and NF-κB pathways, resulting in their activation and the formation of an inflammatory microenvironment containing a complex combination of cytokines and chemokines, which accelerates GC development and progression (174). Our discussion will centre on cytokines, since the role of chemokines in GC has been recently excellently reviewed (185).

A number of DNA polymorphisms in TNF and STAT controlled cytokine genes have been mapped, including IL-1β, IL-1R, -8, -6, -17A, 17F, -22, that may mediate differences in response to chronic *H. pylori* infection and therefore risk for gastric cancer (186–191) and reviewed (183). Specifically, a recent meta-analysis study of 46 publications strengthened the idea that several TNF polymorphisms may be associated with GC risk, particularly TNF-857 in Caucasians and TNF-1031 in Asian populations (192). As a pro-inflammatory cytokine, low-dose chronic TNF secreted by inflammatory cells sustains inflammation, chemokine expression, whilst promoting angiogenesis and inflammation-associated neoplastic progression (193, 194). A recent analysis of patient GC RNA transcript levels has shown that many TNF responsive cell survival genes, (e. g. TRAF2, C-FLIP) are up-regulated favoring a pro-tumoral effect, while pro-apoptotic genes as caspase-3 and TNFR1 are down-regulated during GC development (**Figure 2B**). This suggests that a disequilibrium between the cell death and proliferative processes occurs in GC. Whilst TNF is elevated in *H. pylori*–infected patients (188) its role in GC still remains to be rigorously investigated.

The IL-6 family of cytokines mediates their inflammatory or additional pro-tumor effects through the JAK-STAT signaling pathway by binding to specific transmembrane signal transducers (**Figure 1B** **, 2A**). Signalling through Gp130, a shared receptor element of many of these transducers, is often dysregulated in GC due to elevated levels of IL-6 and IL-11 (84), which activates STAT3 in GC and GC stem cells and is associated with poorer outcomes (195, 196). IL-11, rather than IL-6, appears to show a greater correlation with elevated STAT3 activation in both human and murine GC (197, 198). Another inflammatory cytokine IL-22, which is produced by CAFs, has been shown to enhance the invasive capacity of human GC cells *in vitro* by activating both STAT3 and ERK signaling (184). Elevated IL-11Ra expression is also associated with lymphatic invasion and blood vessel infiltration (199).

Dysregulation of the JAK-STAT pathway more generally has also been documented to contribute to gastric tumorigenesis (60, 200). The role of STAT1 in GC is complex as discussed below and in GC tissues it is regulated in an IFNγ-JAK-STAT-dependent manner (153). Chronic STAT3 activation appears to be pivotal in GC induction (200) and STAT3 has been shown to control the production of many pro-inflammatory cytokines (TNF, IL-1β, IL-6 and IL-22). These factors are known to control the cellular function of immune cells, resulting in a pro-inflammatory state and can also impact multiple functions contributing to nascent tumorigenesis in epithelial cells (201, 202). Active STAT3 is expressed in several established GC cell lines (203) and its inhibition has been shown to mediate their apoptosis. STAT3 is also known to promote the formation of new blood vessels by increasing expression levels of VEGF in GC (60). A recent study also highlighted the role of *H. pylori* in the epigenetic silencing of SOCS1 in GC through hypermethylation of the promotor region, which in addition to inflammatory cytokines, further amplifies JAK-STAT signaling in this cancer (204). Collectively, multiple human GC studies have intimated a role for JAK-STAT signaling, in particular STAT3 in multiple cell types associated with tumorigenic phenomena, such as inflammation, EMT transition and metastasis (60).

### 4.3 JAK-STAT in GC; Lessons From Mouse Models

Transgenic and gene knockout technologies have been utilised to develop murine models of inflammation induced GC, which have been useful to study the influence of cytokines on the progression to tumorigenesis, without the influence of *Helicobacter* (63, 205). Many of these models include mice carrying mutations that result in the overexpression of the JAK-STAT transcription factors, cytokines or modulation of the NF-κB pathway impacting cytokine and STAT protein expression (206–209). Constitutive STAT3 activation promotes gastric tumorigenesis not only in human adenocarcinomas and but also mouse models (200) (**Figure 3B**). The common gp130 subunit heterodimerizes with several IL-6 family co-receptors (**Figure 2A**) (210). One of the best characterized murine models to investigate the role of these cytokines is the *gp130^Y757F/Y757F^* (*gp130^F/F^*) mouse, which carries a homozygous mutation (Y_757_ residue) in the gp130 receptor chain (206, 209). This mutation disrupts the SHP2 and SOCS3 binding site preventing them from shutting down signalling and resulting in the hyperactivation of STAT3, and to a lesser extent STAT1. This in turn leads to upregulation of many STAT3 target genes, including pro-inflammatory cytokines (19, 27, 28). The *gp130^F/F^* mice develop spontaneous gastric adenomas, akin to human intestinal GC (209). Similar to human GC, in this model IL-11 has a more prominent role compared to IL-6 during the progression to GC and a stronger correlation with elevated STAT3 activation. This suggests that for GC, targeting IL-11 rather than IL-6 may be a more beneficial option (198, 211). Excessive STAT3 activation, fuelled and maintained by tumor-associated IL-11 expression, appears to be sufficient to trigger neoplastic behaviour of gastric epithelium without the requirement for additional predisposing genetic alterations (212). This IL-6/IL-11-dependent increase of STAT3 expression also contributes to the development and progression of *H.pylori*-associated GC (213), while loss of STAT3 or STAT1 prevented disease (211). Overall, these animal studies have provided valuable insights into how STAT3-driven inflammation drives GC tumorigenesis and identify IL-11 as a crucial cytokine promoting chronic gastric inflammation and associated tumorigenesis mediated by excessive activation of STAT3 and STAT1 (197, 212).

*H. pylori* or EBV infection in addition to activating STAT3 can result in sustained activation of NF-κB. We have recently shown that mice lacking NF-κB1 (*Nfkb1^-/-^*), a member of the canonical NF-κB signaling pathway develop inflammation-driven invasive gastric adenocarcinoma (208). GC in *Nfkb1^-/-^* mice mimics the histopathology of the human disease and is associated with elevated STAT1, and to a lesser extent STAT3, in the gastric mucosa (208), recapitulating the pathogenic loss-of-function polymorphisms in NF-κB1 associated with human GC (207, 214). Similar to the human disease, GC in this mouse model is associated with elevated pro-inflammatory cytokines and chemokines such as TNF, IL-6 and IFNγ. Consistent with the foregoing discussion there is an earlier onset of GC in *Nfkb1^-/-^*/*gp130^+/F^* mice (207, 208).

As discussed for oral cancer, while STAT1, exerts tumor suppressive activities, by integrating the anti-proliferative and pro-cell death signals elicited by interferons, it can also drive tumor promoting activities in stromal cells. These activities include inducing an immunosuppressive tumor environment by regulating immune checkpoint inhibitor expression, such PD-L1 and PD-L2 the ligands for PD-1, which can be induced in tumors by IFNs, resulting in immune evasion (80). We established a direct link between NF-κB1 loss-of-function and upregulation of STAT1 pro-tumorigenic functions and the immune checkpoint PD-L1 (208). Pertinently, JAK2 is also amplified in human EBV^+^ GC (77) and cellular RNA expression of *Jak2* is abnormally elevated in the gastric mucosa of *Nfkb1^-/-^* mice before overt GC (208). Moreover, the mechanism underlying the regulation of PD1 and PD-L1 in human the EBV^+^ GC subtype has recently been shown to be controlled by the JAK-STAT1-IRF3 signaling axis (153). Therefore, the immune evasion of EBV^+^GC cells could be regulated by this signaling pathway, which may be further investigated using these murine models (215). More recently we showed that IL-6, and IL-22 and the receptor for IL-11 (IL-11Rα) are dispensable for the development of GC in *Nfkb1^-/-^* mice (207). However, the loss of IL-11Rα significantly reduced invasive GC disease and loss of TNF inhibited GC development in *Nfkb1^-/-^* mice but to a lesser extent than complete loss of STAT1 (207). Notably, the loss of either TNF or STAT1 reduced gastric inflammation and PD-L1 expression in the stomach (207). Insights from this model reveal a role for TNF in GC development, identify a role for IL-11Rα in invasive GC disease and uncover a link between elevated TNF levels and aberrant STAT1 activation shaping the gastric immune microenvironment. These findings suggest that inhibition of IL-11/IL-11Rα signaling for example by using IL11-Mutein might have clinical benefit (197, 198, 216). While inhibitors of TNF are readily available compared to STAT1 (currently do not exist), these potential therapies may extend to a broad range of GCs, not only those with *NFKB1* gene polymorphisms, a topic we will discuss in the final section of this review.

## 5 Therapeutic Potential of Targeting the Cytokine and JAK-STAT Pathway

Surgery and chemoradiotherapy/radiotherapy (CRT) are the mainstays for advanced OSCC/HNSCC, because effective and targeted therapies which are still wanting (142, 217). Similarly, the prognosis for advanced GC remains abject due to the poor response to current therapies (chemotherapy, surgery, chemo/radiotherapy) (60, 96, 218, 219). Due to a deficit of targeted therapies, additional or adjunct therapies are warranted to advance treatments for these cancers. Blocking JAK-STAT signaling may be a solution, either as a single therapy, or in combination with other anti-cancer agents. There are multiple potential intervention points for targeting JAK-STAT signaling, including various cytokines, their receptors, STATs, JAKs, SOCS (**Figure 3**) as well as other cytokine signalling pathways, such as TNF. Inhibitors abrogating the JAK-STAT pathway tested in pre-clinical and clinical studies have been recently extensively reviewed (4, 8, 15, 56, 71, 82, 220, 221) and so we will here focus on HNSCC/OSCC and GC.

### 5.1 Targeting STATs

Therapeutic targeting of STAT transcription factors is not without challenges. Transcription factors have been deemed “undruggable” due to their lack of catalytic function (82, 222). While there are many studies using conventional small molecule drugs, unfortunately, both off-target adverse effects and on-target toxicity have tarnished the progression of these compounds and few have transited to clinical trials for solid cancers (4, 56, 60, 71, 72, 82, 220, 221) and even more limited for HNSCC/OSCC and GC (**Table 1**).

**Table 1:** Clinical Trials for JAK-STAT/TNF Pathway modifiers in HNSCC or GC.

Inhibitor/ Modulator	Inhibitor Agent/Adjuvant Therapy	Phase	Clinical Trial	Status (11 Nov. 2021)	Ref/results
STAT3	BBI-608(Napaucasin)/paclitaxel	Ib/II	NCT01325441	Completed ^a^	223
		III	NCT02178956	Completed ^h^	224
STAT3	OPB-111077 - SH2 domain binder	I	NCT01711034	Completed ^a^	225
STAT3	TTI-101/C188-9 – SH2 domain binder	I	NCT03195699	Recruiting ^b^	None
STAT3	AZD9150 (ISIS 481464) – antisense oligo	I/II	NCT01563302	Completed ^c^	226
STAT3	OPB-31121 - SH2 domain binder	I	NCT00955812	Completed ^d^	227
		I	NCT00657176	Unknown ^d^	228
STAT3	OPB-51602 - SH2 domain binder	I	NCT02058017	Terminated ^e^	None
		I	NCT01423903	Completed ^a^	None
		I	NCT01184807	Completed ^d^	None
		I	NCT01867073	Active, not recruiting ^d^	None
STAT3	STAT3 decoy - Oligonucleotide	0	NCT00696176	Completed ^f^	229
STAT3	AZD9150/ anti-PD-L1 (Durvalumab)	Ib/II	NCT02499328	Active, not recruiting ^f^	Submitted-not posted
STAT3	IMX-10 (curcumin/doxorubicin)	I/II	NCT03382340	Recruiting ^d^	None
STAT3	PROTAC (KT-333)	I	NCT05225584	Recruiting ^c^	230
JAK1	Itacitinib (INC039110)/pembrolizumab, anti-PD-1)	Ib	NCT02646748	Active, not recruiting d	None
JAK1/2	AZD-1480	I	NCT01112397	Terminated ^d^	231
		I	NCT01219543	Terminated ^g^	None
	Ruxolitinib	II	NCT03153982	Recruiting ^f^	None
		0	NCT02593929	Withdrawn ^f^	None
IL-6Ra	Tocilizumab (anti-IL-6Ra) / atezolizumab (anti-PD-L1)	II	NCT03708224	Recruiting ^f^	None
	Siltuximab	I/II	NCT00841191	Completed ^d^	232
IRX-2 biologic (Il-1&beta;, Il-2, IL-6, IFN&gamma;, TNF, GMCSF)	IRX-2/ cyclophosphamide, pembrolizumab	I/II	NCT03918499	temporary on hold ^h^	None
	IRX-2/ cyclophosphamide, indomethacin, omeprazole, zinc	II	NCT02609386	Active not recruiting ^i^	None
	IRX-2/ anti-PD-L1 (Durvalumab)	I	NCT03381183	Active not recruiting ^f^	None
	IRX-2/Nivolumab	I	NCT03758781	Active not recruiting ^j^	233
	IRX-2/cyclophosphamide, indomethacin, omeprazole, zinc	II	NCT00210470	Completed ^f^	234
EGFR	Cetuximab/cisplatin/docetaxel/radiation	II	NCT00084318	Completed ^f^	235
	Cetuximab/cisplatin/bortezomib/radiation	I	NCT01445405	Completed ^f^	None
	Cetuximab/pembrolizumab (anti-PD-1)/radiation	II	NCT02707588	Active not recruiting ^f^	236
cIAP1/2	Smac-mimetic (Debio1143)/cisplatin/radiation	I/II	NCT02022098	Active, not recruiting ^f^	237
cIAP1/2	Smac-mimetiAc (Debio1143)/ Cisplatin/IMRT	III	NCT04459715	Recruiting ^f^	None

a Advanced malignancies.

b HNSCC, gastric adenocarcinoma, plus other solid tumor types.

c Advanced cancers or hematological malignancies.

d Advanced cancers and/or solid tumors.

e Locally advanced nasopharyngeal carcinoma

f HNSCC.

g Advanced solid malignancies, with GC in the expansion phase.

h gastric or gastroesophageal junction cancer.

i Squamous carcinoma of the oral cavity. IMRT (Intensity Modulation Radiation Therapy).

j recurrent/metastatic solid tumors including HNSCC.

#### 5.1.1 Targeting STAT1

STAT1 inhibitors are still under development using comparative virtual screening and docking validation (238). An important point to bear in mind is that STAT1 takes part in IFN signaling and thus plays important roles in barrier function and host defence against infections, therefore its inhibition may not be of optimal benefit for translation to the clinic (4, 8, 220).

#### 5.1.2 Targeting STAT3

Because of its strong pro-oncogenic function, most STAT inhibition studies have focused on this transcription factor with the aim of blocking phosphorylation and/or STAT dimerization (221). However, inhibition of STAT3 is also problematic, since it can be activated by several different upstream kinases and development of an inhibitor that specifically targets STAT3 rather than STAT1 to improve therapeutic efficacy remains challenging (15, 56, 239). While a number of STAT3-inhibiting compounds have been developed (4, 71, 221) (**Figure 2A**), they generally have low potency, poor specificity and inappropriate pharmacology constraining their progression into the clinic and approval (71, 82, 239). Newer compounds are being synthesized and evaluated by computational methods to improve the understanding of the STAT3 functional mechanism and aid in the design of STAT3 inhibitors as anti-cancer drugs (239, 240). These consist of direct STAT3 inhibitors (peptides, small molecules, oligonucleotides), indirect inhibitors (JAKs, IL-6, EGFR) or those that can be combined with immunotherapy (e.g. immune checkpoint inhibitors, CAR-T cell therapy, dendritic cell based cancer vaccine and immunostimulatory Toll Ligand Receptor (TLR) agonists) (71).

STAT3 is also a major therapeutic target under investigation for HNSCC/OSCC/GC [reviewed (71, 72, 82)] and while studies are predominantly at the preclinical or early clinical stage, they hold some promise. For example, the STAT3 decoy (double-stranded DNA containing STAT3-binding site) that sequesters dimeric STAT3 away from endogenous targets has been shown to increase apoptotic death and reduce tumor growth in laryngeal squamous cell carcinoma (PCI-37A) (241). This STAT3 decoy is being tested in the clinic, where the expression levels of STAT3 target genes were shown to decrease in HNSCC/OSCC following STAT3 decoy injection (229) (**Table 1** **)**. STAT3 signaling activity can also be attenuated by Stattic, a small molecule STAT inhibitor, which targets the SH2 domain, resulting in the modulation of invasion and migration of OSCC cell lines (242). In nasopharyngeal carcinoma cell lines Stattic has been shown to inhibit cell viability and proliferation, induce apoptosis and enhanced chemo/radio sensitivity (243).

The most successful STAT3 inhibitor to date is napabucasin/BBI-608 which inhibits JAK2 and STAT3 phosphorylation and transcription of target genes (244) (**Figure 2A**; **Table 1**). It received orphan designation from the FDA (2016) for gastric cancer due to early positive results for the phase Ib/II trial (223), however the phase III (BRIGHTER trial) as a second-line treatment in combination with paclitaxel in patients with gastric and pre-treated advanced gastric and gastroesophageal junction (**GEJ**) cancer showed no improvement in overall or progression free survival (224). Subgroup analysis is pending, and this may provide some insight for better patient selection for napabucasin in the future.

The Otsuka Pharmaceutical Co. have developed a number of non-peptide STAT3-SH2 domain inhibitors (OPB-31121, OPB‐111077, OPB‐51602), which have been documented in many phase I trials **(**selected; **Table 1**). OPB-31121 has been shown to reduce proliferation of gastric cancer cells and in a xenograft model, where it was shown to synergize with 5-fluorouracil (5-FU) and cisplatin (245). This inhibitor also interacts with STAT5 and showed antitumor activity in various hematopoietic malignancies (246). However, phase I trials for advanced malignancies were terminated before many participants had been enrolled **(** **Table 1**). On the other hand, it has undergone successful phase I/II trials for advanced hepatocellular carcinoma (ClinicalTrials.gov #NCT01406574) and for solid cancers (**Table 1**), (227) and is currently in a phase III trial in combination with 5-FU, Leucovorin and Irinotecan (FOLFIRI) for metastatic colorectal cancer (ClinicalTrials.gov #NCT03522649) and in a completed but yet to be reported trial with nab-paclitaxel and gemcitabine for metastatic pancreatic ductal carcinoma (CanStem11P trial, ClinicalTrials.gov #NCT02993731). OPB‐111077, another novel inhibitor of STAT3, also exhibits promising anti-cancer activity in patients with diffuse large B‐cell lymphoma (DLBCL) and modest efficacy was observed against other tumors, including GC, when given as a monotherapy (225). Phase I studies with OPB‐51602, have been disappointing in hematological malignancies (247) and locally advanced nasopharyngeal carcinoma but are currently in trial for solid cancers (**Table 1**).

The small-molecule competitive STAT3 inhibitor TTI-101 (formerly C188-9) developed by Tvardi, targets the pY-peptide binding site within the SH2 domain (**Figure 2A**) to prevent phosphorylation, homodimerization, nuclear translocation and transcriptional activation (221). TTI-101 has been shown to inhibit cytokine-stimulated pSTAT3 and reduce constitutive pSTAT3 activity in multiple HNSCC cell lines, including the radioresistant HNSCC cell line UM-SCC-17B in a xenograft model. In this system, TTI-101 prevented tumor growth by modulating many STAT3-regulated genes affecting oncogenesis and radio-resistance, as well as radio-resistance genes regulated by STAT1, due to its potent activity against not only STAT3 but also STAT1 (248). More recent studies have shown that TTI-101 can be given orally and without toxicity (249) and this STAT3 inhibitor is currently being trialled as a monotherapy for solid tumors including HNSCC and GC (**Table 1**).

AZD9150 is a second generation antisense oligonucleotide targetting the 3’ untranslated region of STAT3. It inhibits mRNA translation and has shown efficacy in pre-clinical models (226), where it inhibited tumor growth and expression of STAT3 downstream target genes. It has also shown efficacy in phase I/Ib clinical trials for both lung cancer and lymphoma (142, 226, 250). A Phase I/II dose-expansion study for the treatment of patients with advanced cancers, DLBCL and other advanced lymphomas (ClinicalTrials.gov NCT01563302) has been completed but no results are available. Another phase II trial using AZD9150 in advanced solid tumors including metastatic HNSCC as a monotherapy or combined with MED14736/Durvalumab immunotherapy (anti-PD-L1) is currently underway (ClinicalTrials.gov #NCT02499328) **(** **Table 1**).

Another method to target STAT3, involves the use of double-stranded “decoy” oligonucleotides, corresponding to STAT3 response elements, such as those present in the c-fos promoter. An analogous STAT3 decoy has been shown to block binding of STAT3 and when used to treat HNCSS cell lines inhibited proliferation and reduced STAT3-mediated gene expression (241). A phase 0 trial of this STAT3 decoy (ClinicalTrials.gov #NCT00696176, **Table 1**) determined that intra-tumoral administration in HNSCC patients inhibited STAT3 driven gene expression. In addition, a kinetic study involving a xenograft model showed that administration of the STAT3 decoy, but not the mutant control decoy, decreased expression of STAT3 target genes (Bcl-xL and/or Cyclin D1) (229).

Finally, YHO-1701 a novel quinoline- carboxamide derivative of STX-0119 and a non-peptide SH2 domain STAT3 inhibitor has been shown to inhibit the SH2 binding to a p-Tyr peptide more potently than the original STX-0119 in biochemical assays (251). YHO-1701 also exhibited strong activity in abrogating STAT3 signaling in the human OSCC cell line SAS, by inhibiting STAT3 dimerization and also suppressing STAT3 promoter activity. In addition, YHO-1701 showed anti-tumor effects in SAS xenograft models in combination with sorafenib producing an anti-proliferative and synergistic effect in SAS OSCC cells, justifying future clinical follow-up (252). Overall, the studies evaluating STAT3 inhibitors for OSCC/HNSCC and GC at pre-clinical and early clinical trial stages suggest that it is unlikely that they will be used as a monotherapy. However, there is still the possibility of using them as adjunct therapy, which will be discussed later in this section.

### 5.2 Targeting JAKs

The cornerstone of JAK-STAT signaling inhibition are the JAK family members (**Figure 1**) and these have been extensively studied for inflammatory diseases such as rheumatoid arthritis (253). A variety of JAK inhibitor compounds are available at the clinical or pre-clinical stage as orally delivered small molecules targeting the ATP-binding site of the JAK protein, preventing their phosphorylation and the subsequent phosphorylation of STATs thus diminishing JAK activity and nuclear signaling (59, 82). Since JAKs are upstream of STATs and therefore might also interfere with other mechanisms involved in cancer progression this could explain a perceived improved efficacy of JAK inhibitors and the popularity of Tofacitinib (JAK1/JAK3 inhibitor) and Baricitinib (JAK1/JAK2 inhibitor) for the treatment of inflammatory conditions (4, 8, 59, 60, 82, 253, 254). As an interesting aside, a relatively selective JAK1 inhibitor, Oclacitinib, is used to treat dermatitis in dogs and lacks the side effects that most JAK inhibitors have in humans (255). Ruxolitinib, a JAK1/JAK2 inhibitor is FDA approved for the bone marrow cancer myelofibrosis, polycythemia vera and topically for atopic dermatitis (59, 71, 82, 254) and has also shown efficacy in solid cancers, for example in HNSCC cell lines (256). However, an early phase 0/I trial with ruxolitinib as a monotherapy administered prior to surgical resection for operable HNSCC was withdrawn due to adverse events (ClinicalTrials.gov #NCT02593929), but a phase II trial is currently recruiting (ClinicalTrials.gov #NCT03153982) (**Table 1**). Trials have also recently commenced for a range of other JAK inhibitors for solid malignancies, but with limited success (4, 59, 71, 254). For example, AZD140 (JAK1/2 inhibitor) abrogated STAT3 activation and HNSCC tumor growth in a patient-derived xenograft preclinical model (257), but a study to assess the safety and tolerability of AZD140 as an oral monotherapy in patients with solid tumors (ClinicalTrials.gov #NCT01112397) (231) and a phase I study including GC in the expansion phase (ClinicalTrials.gov #NCT01219543) have both been terminated due to a decision to stop development of this JAK inhibitor (**Table 1**). It is noteworthy that JAK inhibitors are not specific for each JAK and there are issues with off-target effects (253), thus many clinical trials for solid tumors have been terminated (59) (**Table 1**). It is clear that additional JAK inhibitor specificity is necessary to optimize future therapeutic applications. In addition, adverse events associated with the wide-ranging immunological effects of JAKs (258), have been reported and thus the future of JAK monotherapy applications may be limited (15, 253). However, novel PROTACs targeting the Janus kinase family (JAK1, JAK2, JAK3 and TYK2) have been recently described which may overcome these shortfalls (259, 260).

### 5.3 Inhibiting SOCS

Since SOCS proteins negatively regulate JAK-STAT cytokine signaling, they can play major roles in limiting the evolution and subsequent progression of tumorigenesis. Atypical SOCS1 and SOCS3 expression in established tumor cell lines and also at advanced clinical stages of cancer is considerably variable (261). However, SOCS1 mediated negative signaling feedback has been shown to be important for inflammation reduction (262) and also to limit nascent tumor growth (263). Similarly, epigenetic inactivation due to CpG methylation in SOCS1 is frequently linked to several human solid cancers including GC and may be involved in its development, progression and even metastasis, since reduced expression of SOCS1 was associated with lymph node metastasis and more severe GC tumor stage (264). In addition, SOCS1 and SOCS3 can control the development and activation status of immune cells in tumorigenesis but their exact roles in this process are still unclear (261, 265, 266). Thus, SOCS-based targeting is in its infancy (267).

Since SOCS seem to inhibit tumor progression different strategies have been used to either increase or mimic SOCS activity. For example, SOCS1 and SOCS3 expressing oncolytic adenovirus (CN305 (AdCN305)-SOCS3) or recombinant, cell-permeable proteins has been shown to efficiently control aberrant STAT signaling in hepatocellular carcinoma (268). A SOCS1 peptide mimetic (containing the KIR domain) which acts as a pseudosubstrate for JAK1, JAK2, TYK2 and JAK3 and is able to activate the endogenous SOCS1 protein has also been pursued for therapeutic applications. Their safety and efficacy particularly in comparison to JAK inhibitors is yet to be fully evaluated (263).

### 5.4 Targeting Cytokines

An emerging opportunity for cancer therapy to circumvent the hazards of targeting intracellular signaling molecules such JAKs, or STATs is through pharmacologic inhibition of proteins that activate them, such as pro-inflammatory cytokines (71, 73, 198, 269). Since cytokines are major drivers of several autoimmune/inflammatory diseases, it is not surprising that cytokine inhibition has revolutionized therapy for these disorders, particularly with monoclonal antibodies targeting TNF, IL-1, IL-2 and IL-6 (116). Cytokine-based immunotherapy for cancer treatment is encouraging, since cytokines are able to regulate the host immune response to directly induce cancer cell death (270). Recently this has led to an interest in the efficacy of cytokine-based drugs, as single agents or in a combinative approach with other immunotherapy drugs to improve the anti-tumor properties of cytokines. Newer second-generation drugs aimed at improving cytokine activity in the tumour microenvironment or towards the desired effector immune cells, however, are yet to reach the clinic (270). In this review we will focus on modulation of IL-6 and IL-11 which signal through the JAK-STAT pathway (**Figures 1** **, 2A**) and TNF, which signals through the TNF family (**Figure 2B**). In addition, we discuss how antagonizing the activity of these cytokines has merit in overcoming toxicity to improve tolerance of cancer immunotherapy, including for OSCC and GC.

#### 5.4.1 Targeting IL-6 and IL-11

Many therapies target cytokines or their receptors (**Figure 2**) and to date, much focus has been placed on antagonizing the activity of IL-6, since elevated levels of this cytokine can mediate hyperstimulation of JAK-STAT signaling through the gp130 receptor (197, 269, 271). Antagonistic monoclonal antibodies such as tocilizumab, against IL-6R, and siltuximab against IL-6 (**Figure 2A**), were originally developed to treat inflammatory diseases, and tocilizumab was granted an emergency use authorization in the US for COVID. Other IL-6 targeting agents are also in development: sarilumab, ALX-0061, olokizumab, sirukinumab and clazakinumab (82, 269). Inhibitors of IL-6 and IL-6Rs are now undergoing clinical trials for solid cancers: ovarian, renal, prostate, lung, melanoma, pancreatic and breast (71, 82, 272, 273). However, the IL-6 ligand antibody (siltuximab/CNTO-28) trial for prostate cancer, did not improve clinical outcomes (274).

A phase I/II trail with siltuximab for select solid advanced solid tumors, including HNSCC with KRAS mutations, while well tolerated was without clinical activity (232) (**Table 1**). Our preclinical studies in a GC cancer model driven by the absence of NF-κB1, have shown that genetic deletion of IL-6 is dispensable for the development of GC, with a minor role for IL-6 at the early stages of gastric dysplasia (207). Studies antagonizing the activity of IL-6 for HNSCC and GC are not yet forthcoming. However, as indicated earlier in this review, IL-6 trans-signalling through a soluble form of the IL-6Rα (81) is involved in inflammation driven tumor response. This signalling can be selectively inhibited using a soluble form of gp130 (sgp130) fused to an IgG Fc fragment, as sgp130Fc (olamkicept/FE 999301/TJ301) (**Figure 2A**). This first-in-class decoy protein exclusively blocks IL-6 proinflammatory trans-signaling and has shown clinical efficacy in early phase IIa trials for ulcerative colitis/inflammatory bowel disease (IBD) without immunosuppression but with p-STAT3 reduction in the inflamed IBD mucosa [ClinicalTrials.gov #NCT03235752), (275). However, sgp130Fc is yet to be trialled in solid tumors. Other designer mutants to modulate IL-6 signaling are in early development (197)].

Another IL-6 family cytokine, IL-11 also signals through gp130 to activate JAKs/STAT3 (**Figure 2A**). IL-11 is known to be a pleiotropic in character and to play a role in hematopoiesis, adipogenesis and platelet maturation (197). In this capacity, IL-11 predominantly acts as an anti-inflammatory cytokine, with potential to increase platelet counts in chronic myelogenous leukemia. More recently its role in several inflammation driven cancers, such as gastrointestinal cancers has been identified (197, 276), perhaps providing a link between inflammation and cancer. IL-11 can increase the oncogenic properties of cells, including, cancer cell proliferation and survival (197, 277). For example, elevated expression has been linked to several cancers, including GC, where IL-11 levels are elevated in preclinical models of GC (277). These findings support a role for IL-11/IL-11Rα signaling inhibition as an emerging therapeutic opportunity for multiple cancers, including GC, perhaps through implementation of IL11-Mutein, an antagonist of IL11Rα (216). However, our understanding of how IL-11 impacts the initiation and progression cancers is still limited and no agent inhibiting IL-11 is currently approved for the treatment of solid cancers (197).

#### 5.4.2 Targeting TNFR Pathway

The master proinflammatory cytokine TNF can serve as either therapeutic target or agent. As a therapeutic target, it can be inhibited with well established anti-TNF biologics, including etanercept, infliximab, adalimumab, golimumab, and certolizumab pegol (278) for inflammatory diseases (116). As discussed in this review, TNF is also important at many stages in OSCC (94, 136) and GC (63), therefore targeting the TNFR signaling pathway may be an effective preventive or therapeutic strategy for solid cancers. Indeed, the use of anti-TNF drugs to treat cancer has a long and interesting history (58, 85). Some early preclinical studies were encouraging in this regard; for example blocking TNF with golimumab reduced tumor growth, angiogenesis and metastasis of OSCC in a murine model of orthotopic human OSCC (279), and antagonizing TNF reduced oral cancer proliferation and cytokine production in mice with 4NQO induced oral cancer (135). TNF/TNFR1 signaling also been shown to promote gastric tumorigenesis in the *Gan* mouse model, in which transgenesis activates both canonical Wnt signaling and the COX-2/PGE_2_ pathway (280). Clinical studies with anti-TNF therapy are yet to be published for OSCC or GC (273).

Another approach to enlist TNF signaling are the smac-mimetics, a drug class that antagonises Inhibitor of APoptosis proteins (IAPs: XIAP, cIAP1, and cIAP2). Several small molecule smac-mimetic compounds have been developed and many have been or are currently in clinical trials, including Debio 1143/AT-406/SM-406, LCL161, APG1387, BI 891065 and ASTX660 (281, 282). These promote the proteasomal degradation of the cIAPs, resulting in TNF production in a subset of ‘responsive ‘ cells (283–285). IAP overexpression is associated with tumorigenesis, chemoresistance and poor prognosis (281, 286, 287) and the IAPs also positively (canonical) and negatively (non-canonical) regulate NF-κB signalling pathway (281) (**Figure 2B**). We, and others, have shown that smac-mimetics function by instigating auto-ubiquitylation and proteasomal degradation of cIAP1 and cIAP2 (284, 285, 288, 289), which in the presence of a stimulus such as TNF, chemotherapy or TLR ligand, results in the formation of a RIPK1/caspase-8 complex, followed by caspase-8 cleavage and the induction of tumor cell death (281, 285, 290, 291). A number of smac-mimetic compounds have been developed and due to their limited anti-cancer activities when used as single agents are now being tested in combination with immunotherapy and/or chemotherapy (6, 281, 282). For example, a trial reported in 2020, using Debio 1143 in combination with chemo-radiotherapy was the first drug combination in more than three decades to significantly improve overall survival in high risk HNSCC (includes OSCC) patients (ClinicalTrials.gov NCT02022098) (237) and a phase III trial is currently recruiting (ClinicalTrials.gov NCT04459715) (**Table 1** **)**.

#### 5.4.3 Immune Checkpoint Inhibitors Coupled With Cytokine, JAK/STAT Inhibitors

Immune checkpoint dysregulation to escape from immune system surveillance is used by a number of cancers including HNSCC/OSCC and GC to allow their development. Immune checkpoint inhibitor therapy (anti- CTLA-4, PD-1 or PD-L1 antibodies) boosts the cytotoxic activity of tumour antigen-specific T cells and has revolutionized treatment and increased survival for a subset of cancers (292, 293). Boosting the immune response with anti-PD-1 for both HNSCC/OSCC (96, 294) and GC (215, 295), while proven for subsets of patients is not without challenges, including patient selection, bio-markers and adverse events (296, 297). Therapy for OSCC and GC is changing, and pre-clinical studies suggest that JAK-STAT signaling could play a role in regulating the activity of Immune Checkpoint Inhibitor (ICI) therapies (71, 152, 153, 295). The FDA has given approval for the use of ICI therapies for the MSI sub-type of GC (with EBV^+^ also a subtype which might benefit) (77). These subtypes are generally more immunogenic due to high mutational burden and ICI exploits this leading to generally better outcomes (215). However, since IFNγ is a major driver of JAK-STAT1 associated PD-L1 expression (ligand for the immune-checkpoint PD-1), JAK-STAT therapies, which have the potential to block IFNγ signalling, may result in reduced PD-L1 expression, thereby diminishing the effects of ICI. However, it may be possible to circumvent this since it was recently reported that JAK2 inhibitors in combination with ICI had better therapeutic responses, by blocking JAK signaling and repressing the effects on PD-L1 (73). These considerations are starting to focus attention on the immunological context of GC and particularly STAT1 expression as an immunotherapy biomarker (56, 73, 215, 298).

These new advances are exemplified by a new era of personalized therapy in the IMMUNOGAST phase II Trial (ClinicalTrials.gov #NCT04739202) for recurrent GC based on TCGA sub-type and trialling immunotherapy (anti-PD-L1, atezolizumab) plus ipatasertib (AKT inhibitor) for EBV^+^ GC, which exhibits amplified JAK2, ErbB2, PD-L1 and PD-L2 (**Table 1**). Similarly, for advanced solid cancers, including HNSCC, an active phase 1b trial combines pembrolizumab (anti-PD1) with the JAK1 inhibitor itacitinib (INCB039110) (ClinicalTrials.gov #NCT02646748) (**Table 1**; **Figure 2A**). APROPOS, a phase II clinical trial for atezolizumab (anti-PD-L1) in combination with tocilizumab to modulate T cell infiltration as a pre-surgery immunotherapy (ClinicalTrials.gov identifier NCT03708224) is currently recruiting for HNSCC (**Table 1**). Recent studies also highlight the potential for smac-mimetics specifically in combination with ICI and CAR-T-cell therapies (299–301).

Cytokine therapy is challenging because of their high degree of pleiotropism, resulting in many adverse effects. However, their real value in cancer therapy, particularly for oral and gastric cancers may be in enhancing the safety of immunotherapies. Thus, ICI or CAR-T cell therapy can result in inflammatory toxicities, such as immune-related adverse events (irAEs) or cytokine release syndrome (CRS). These are life threatening systemic diseases affecting multiple organs (302, 303). By perturbing the inhibitory control processes of the immune system, ipilimumab (anti-CTLA-4), atezolizumab (anti-PD-L1), or nivolumab/pembrolizumab (anti-PD-1) (293) unleash immune cells potentially resulting in the destruction of healthy tissues, limiting therapy duration and otherwise durable remission (302). Serious irAEs manifest with increased mononuclear cells and elevated levels of inflammatory cytokines, including IL-6 and TNF (302). In this context, treatment of advanced GC with nivolumab has been associated with liver injury accompanied with elevated TNF levels (14), highlighting an opportunity for anti-TNF to be used in conjunction with ICIT (193). A recent review on the topic suggests that short course TNF inhibitors are safe irAE treatment for cancer patients undergoing ICI therapy (304). However, the safety profile for long-term TNF inhibitor use for irAEs is lacking and further clinical studies that directly assess the effect of TNF inhibitor treatment on ICI efficacy are required. Data from preclinical studies hint that TNF neutralization in combination with ICIs reduces clinical irAE and improves antitumor efficacy in tumor models (305).

### 5.5 PROTAC Technology a New Therapeutic Avenue for JAK-STAT Pathway Inhibition

Improvements in selectivity and prevention of drug resistance in cancer treatment may be achievable with a novel approach using Proteolysis Targeting Chimeric (PROTAC) technology developed by the Crews and Deshaies labs (306, 307). In one approach, the ubiquitin-proteasome system (UPS) is hijacked for targeted protein degradation through hetero-functional molecules linking the target protein ligand to an E3 ubiquitin ligase (E3) by a functional linker, resulting in the continuous and rapid deletion of the target protein (181, 308–312). PROTACs have been developed targeting proteins important in cancer growth such as BTK (Bruton’s tyrosine kinase), FAK (Focal adhesion kinase), CDK’s (Cyclin-dependent kinases), Bromodomain-containing protein 4 (BRD4), Mcl-1, MDM2 (312–314) for haematological malignancies (315) and solid cancers, including a photo-controlled BRD4 PROTAC for tongue squamous carcinoma (314).

To date only two orally available PROTACs have reached phase II trials using CRBN (cereblon) as the E3 ligase (312). These PROTACs are showing promising results for prostate (ARV-110) and breast (ARV-471) cancers, targeting the androgen or oestrogen receptors respectively (312, 313, 316). PROTACs are also in clinical development for Bcl-x_L_ (ClinicalTrials.gov. NCT04886622) using an alternative E3 ligase, VHL (von Hippel-Lindau) for both hematologic and solid tumours (312). The success of PROTACs for targeted protein degradation has also progressed from proteasome strategy to harness additional cellular degradation pathways (317). Pertinently, newly emerging degradation technologies are also expanding the scope of PROTACs (312, 318, 319) and their development looks likely to upend the viewpoint that transcription factors are “undruggable” and bring proteins such as those within the NF-κB (230) and JAK-STAT (318) signaling pathways into the druggable orbit.

Despite being considered as a therapeutic target for cancers, attempts to target STAT3 as we have discussed have not met with much success, due to poor target specificity and ineffectiveness in blocking the transcriptional activity of the monomeric form (82, 222, 310, 320). However, STAT3 is an example of the application of PROTAC technology towards the development of small molecule STAT3 inhibitors (306, 310, 320, 321). SD-36, the first described STAT3 PROTAC was designed and developed by the Wang lab using the cell permeable STAT3 SH2 domain and transcriptional inhibitor, SI-109, attached to an E3 ligase analogue of the CRBN ligand lenalidomide (320, 322, 323). SD-36 selectively degraded STAT3 (**Figure 2**), including mutated STAT3 proteins over other STAT proteins, even though they share a conserved SH2 domain (323) in various leukemia, lymphoma (323) and glioma cell lines (324). Functionally in certain leukemia and lymphoma cell lines SD-36 inhibited STAT3 dimerization, DNA binding and target gene activity (323). Prolonged tumor regression in mice xenografted with leukemia, lymphoma cell lines *in vivo* was also achieved with SD-36 and was well tolerated, bringing this PROTAC into the realm of possible therapeutic utility (323). While, SD-36 is yet to enter clinical trials, excitingly the STAT3 targeting PROTAC developed by Kymera (KT-333, **Figure 2**) (321) is recruiting for a first in human phase I trial (Feb 04, 2022, Clinical Trials.gov. NCT05225584) for leukemia, lymphoma and non-specified solid tumors (**Table 1**). Similar to SD-36, KT-333 showed selective degradation of STAT3 over other STAT family members, apoptosis upon treatment of tumor cells, depletion of STAT3 and down-regulation of STAT3 target proteins (321). In pre-clinical *in vivo* xenograft studies KT-333, similar to SD-36 showed a dose dependent growth suppression of hematologic tumors and was well tolerated (321). The development and early clinical trials of PROTACs is an encouraging next phase in the search for STAT3 inhibitors that may yet provide clinical benefit for STAT3-driven cancers such as gastric and oral cancers.

Specific inhibition of select JAK family members is a therapeutic challenge, due to the sequence similarity within pivotal catalytic domains such as the ATP binding site. PROTAC drugs, which destroy their target, are not necessarily constrained to these conserved domains and therefore open up the possibility of specifically targeting JAK family members. Efforts are already underway to exploit this with the design of the first of several series of heterobifunctional PROTAC compounds targeting the JAK family (JAK1, JAK2, JAK3 and TYK2), of proximal membrane bound proteins (259, 260, 325). Solving the structure of JAK inhibitors (e.g. Pyrimidine 1, Quinoxaline 2 or modification of ruxolitinib or baricitinib) bound to the JAK2 tyrosine kinase domain enabled the design and optimization of a series JAK PROTACS t capable of inducing proteosomal degradation of JAK1 and JAK2 (260, 325). The PROTACs capable of removing all possible functions of JAK signaling, rather than just the catalytic activity traditionally associated with JAK inhibitors themselves (259, 325). The most advanced of the JAK inhibitors are series of cereblon (CRBN)-directed JAK PROTACs linked to derivatives of the JAK inhibitors ruxolitinib or baricitinib tested in a panel of leukemia/lymphoma cell lines and xenograft models of kinase-driven acute lymphoblastic leukemia (325). These early studies highlight the potential of JAK-directed protein degradation as a therapeutic approach in JAK-STAT mediated diseases, however they are yet to be tested *in vivo* for solid cancer models or clinically for any disease. We speculate that PROTAC development may extend to the targeting of other JAK-STAT related proteins, such as cytokines (**Figure 2**) and that they may be beneficial for the treatment of not only hematologic malignancies but also extend to solid cancers including oral and gastric cancer, diseases yet to be explored both in pre-clinical models and in the clinic. In conclusion, PROTACS are likely to become a welcome addition to the already available armoury of other targeted drugs and chem-radio therapy available for solid cancers.

### 5.6 Other Mechanisms of Modulating the JAK-STAT Signaling Pathway

Tumor-induced immune suppression is common in patients with advanced malignancies, including HNSCC and GC. Strategies for relieving immunosuppression in HNSCC to restore anti-tumor immune functions, include the neoadjuvant IRX-2, derived from stimulating human PBMCs with phytohemagglutinin (326). IRX-2 contains a cocktail of cytokines including IL-2, IL-1β IL-6, IL-8, TNFα, GM-CSF, and IFNγ (**Table 1**). *In vitro* studies have shown that IRX-2, enhances DC maturation, T cell activation, and NK cell stimulation, to overcome tumor-mediated immunosuppression by activating the tumor environment (326). In clinical settings, IRX-2 administered with chemotherapy promotes anticancer immune responses (234). For example, in a phase IIa trial, patients with previously untreated HNSCC, IRX-2 increased tumor infiltration of T cells, B cells, and DCs and was associated with tumor reduction and prolonged patient survival (**Table 1**). Follow-up and data analysis are under way in a multi-center, randomized, phase IIbINSPIRE trial evaluating the IRX-2 regimen as a stand-alone therapy for activating the immune system to recognize and specifically destroy OSCC tumors (ClinicalTrials.gov #NCT02609386). The IRX-2 regime combined with immune checkpoint therapy is also being trialled for HNSCC and other solid tumors (ClinicalTrials.gov #NCT03758781) (233) (**Table 1**).

One of the few natural compounds tested for treating tumorigenesis is the polyphenol curcumin (diferuloylmethane), the yellow pigment found in turmeric. This natural compound targets many signaling pathways and has been shown to have multiple anti-inflammatory and anti-tumor qualities including anti-tumor metastatic activities through a variety of mechanisms (327). High dose curcumin (30-100mg/kg per day) reduced the tumor inducing potential of the carcinogen 4NQO when administered over the same period in a rat model of OSCC, which was associated with downregulation of STAT3 (327). Oral cancer patients given the curcumin containing drug APG-157, which also contains several other polyphenolic compounds, showed a reduction of the inflammatory cytokines IL-1β, IL-6, and IL-8 in salivary fluid, accompanied with elevated immune T cells in the tumor tissue, suggesting its potential use in combination with immunotherapy (328). Further studies in GC have suggested that curcumin can prevent GC cancer cell proliferation, invasion, metastasis, and angiogenesis, reviewed in (329). However, it is difficult to extrapolate from these studies into curcumin consumption due to its various effects on multiple cellular and molecular mechanisms. In addition, it is evident from the current and expanding treatment strategies discussed for both oral (142) and gastric cancer that an appreciation of patient/tumor genetics, the tumor microenvironment and possibly the microbiome also need to be considered for further optimized treatment strategies.

## 6 Conclusions

JAK-STAT/cytokine signaling pathways have been comprehensively studied due to their pivotal roles in both inflammation and many disease conditions including cancer. The core JAK unit activating the transcription factor STAT element is triggered by a variety of receptors and activates a wide range of downstream targets. Deregulated JAK-STAT signalling and particularly dysregulation of JAK2, STAT3, Il-6 and TNF are commonly associated with driving inflammation and carcinogenesis, including both OSCC and GC. Therefore, the targeting of these signaling pathways with inhibitors holds potential as clinical interventions for these cancers. There are a number of specific inhibitors of the JAK-STAT/cytokine cascade that have already been approved by the FDA for inflammatory conditions and which are being investigated for efficacy in clinical trials for haematopoietic malignancies and a few select solid tumors. However, despite promising clinical outcomes in inflammatory immune diseases and in tumor animal studies, toxicity issues have hindered adoption of these therapeutic approaches in the clinic. Investigating other avenues, such as the addition of cytokine blockade or smac-mimetics may provide synergy for more efficacious inhibition of OSCC and GC tumorigenic progression. More recent advances such as blocking JAK-STAT signaling with small molecules such as PROTACs may provide even better therapeutic precision in conjunction with other anti-cancer agents already in the clinic such as immunotherapy.

## Author Contributions

LO’R wrote the original review and YN, JL and JS reviewed edited and assisted with figures. All authors have read and agreed to the published version of the manuscript.

## Funding

This review was in part funded by a Cancer Council Victoria Grant-in-Aid project, grant number 1161400 (to LO’R), a Cancer Australia and a Cancer Council New South Wales project grant number G20-13 (to LO’R), the Victorian State Government (OIS grant), NHMRC fellowships to (to JS) NHMRC 1107149 & 1195038 and National Natural Science Foundation of China Grant Nos. 81772880, 82173159 (to YN), a Key Research and Development Project (Jiangsu Province) No.BE2020628 (to YN).

## Conflict of Interest

The authors declare that the research was conducted in the absence of any commercial or financial relationships that could be construed as a potential conflict of interest.

## Publisher’s Note

All claims expressed in this article are solely those of the authors and do not necessarily represent those of their affiliated organizations, or those of the publisher, the editors and the reviewers. Any product that may be evaluated in this article, or claim that may be made by its manufacturer, is not guaranteed or endorsed by the publisher.
